# Supplementary material for: An inorganic-blended p-type semiconductor with robust electrical and mechanical properties
Source: Nat Commun. 2024 May 24;15:4440. doi: 10.1038/s41467-024-48628-z (PMC11126573; doi:10.1038/s41467-024-48628-z)
Supplement: Supplementary file 1 — Supplementary Information [file 41467_2024_48628_MOESM1_ESM.pdf]

An inorganic-blended p-type semiconductor with robust electrical and mechanical properties

You Meng[1,2,†], Weijun Wang[1,†], Rong Fan[3,4,†], Zhengxun Lai[1], Wei Wang[1], Dengji Li[1], Xiaocui Li[3], Quan Quan[1], Pengshan Xie[1], Dong Chen[1], He Shao[1], Bowen Li[1], Zenghui Wu[1], Zhe Yang[5], SenPo Yip[6], Chun-Yuen Wong[5,*], Yang Lu[4,7,*], Johnny C. Ho[1,2,6,*]

[1]*Department of Materials Science and Engineering, City University of Hong Kong, Kowloon 999077, Hong Kong SAR*

[2]*State Key Laboratory of Terahertz and Millimeter Waves, City University of Hong Kong, Kowloon 999077, Hong Kong SAR*

[3]*Department of Mechanical Engineering, City University of Hong Kong, Kowloon 999077, Hong Kong SAR*

[4]*Chengdu Research Institute, City University of Hong Kong, Chengdu 610200, China*

[5]*Department of Chemistry, City University of Hong Kong, Hong Kong SAR 999077, P.R. China.*

[6]*Institute for Materials Chemistry and Engineering, Kyushu University, Fukuoka 816 8580, Japan*

[7]*Department of Mechanical Engineering, The University of Hong Kong, Kowloon 999077, Hong Kong SAR*

†*These authors contributed equally to this work.*

[*]*Corresponding author: Johnny C. Ho (johnnyho@cityu.edu.hk); Yang Lu (ylu1@hku.hk); Chun-Yuen Wong (acywong@cityu.edu.hk)*

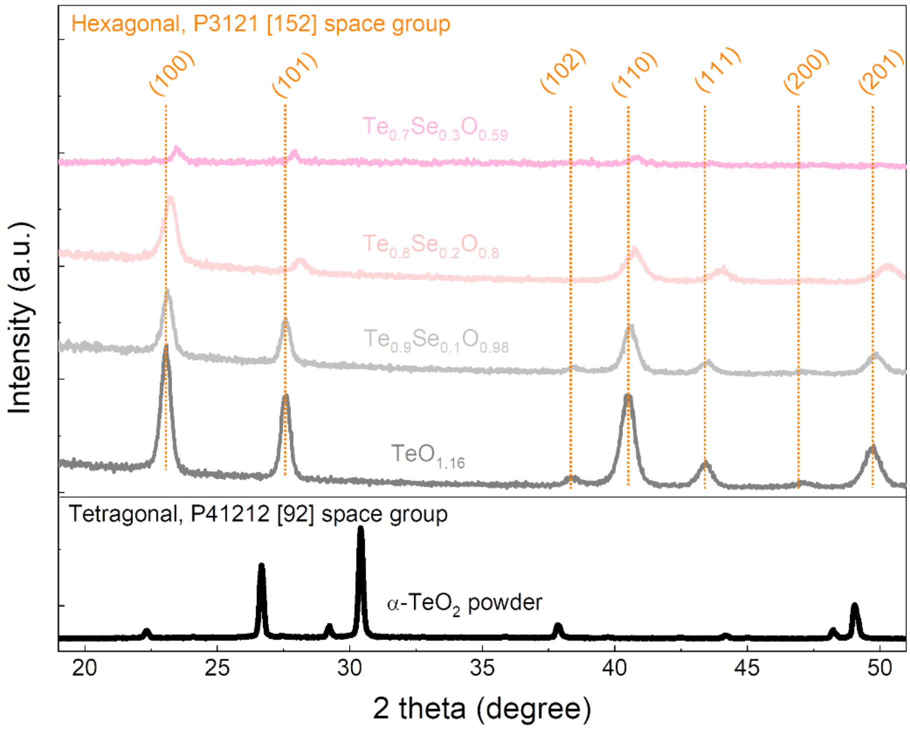

Supplementary Fig. 1. GIXRD patterns of TeSeO thin films with different compositions and XRD pattern of the α-TeO_2 powder. All the diffraction peaks agree with the typical hexagonal crystal system with $P3_121$ [152] space group composed of chalcogen chains along the c axis. The diffraction peak positions shift slightly to higher angles with increasing Se content, indicating the decrease of the lattice constant. This finding could result from the Se substitution in Te sites, in which the Se atom has a relatively smaller radius of 0.14 nm than that of the Te atom (0.16 nm). At the same time, the increased full width at half-maximum of the diffraction peaks also reveals the suppressed material crystallinity.

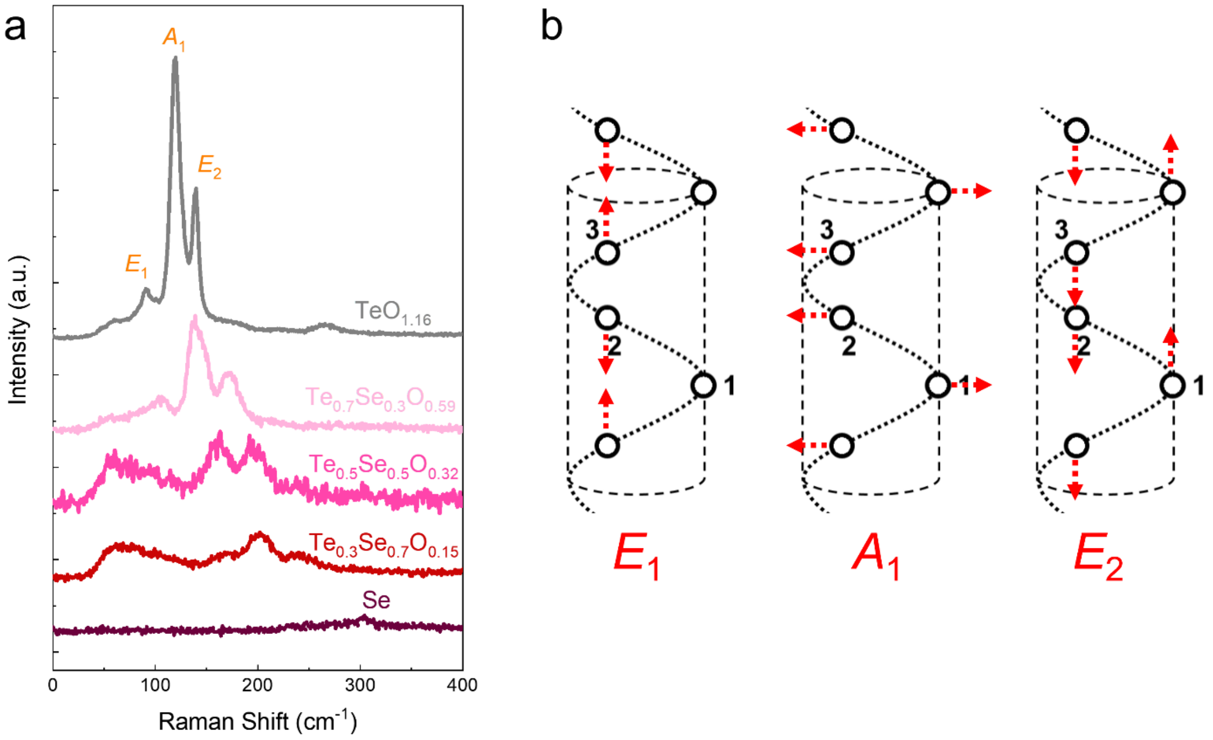

Supplementary Fig. 2. (a) Raman studies of TeSeO thin films with different compositions, where three first-order Raman active modes located at 90 cm^{-1} (E_1 transverse (TO) phonon mode), 118 cm^{-1} (A_1 mode), and 138 cm^{-1} (E_2 mode) were identified. (b) Schematic vibration patterns of the Raman modes of E_1, A_1, and E_2 in chiral-chain Te or Se materials. The E_1 and E_2 modes represent bond-bending and bond-stretching with a larger admixture, respectively, whereas the A_1 mode is caused by chain expansion in the basal plane.

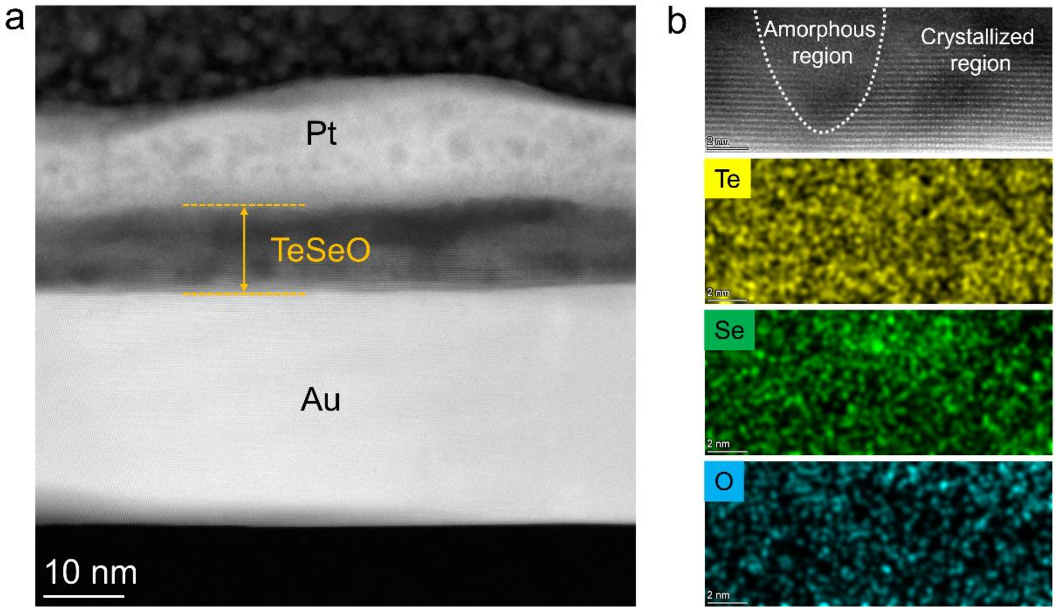

Supplementary Fig. 3. (a) Cross-sectional STEM-HAADF image and (b) EDS mapping of the TeSeO film. The distributions of Te, Se, and O signals are relatively uniform without noticeable element segregation.

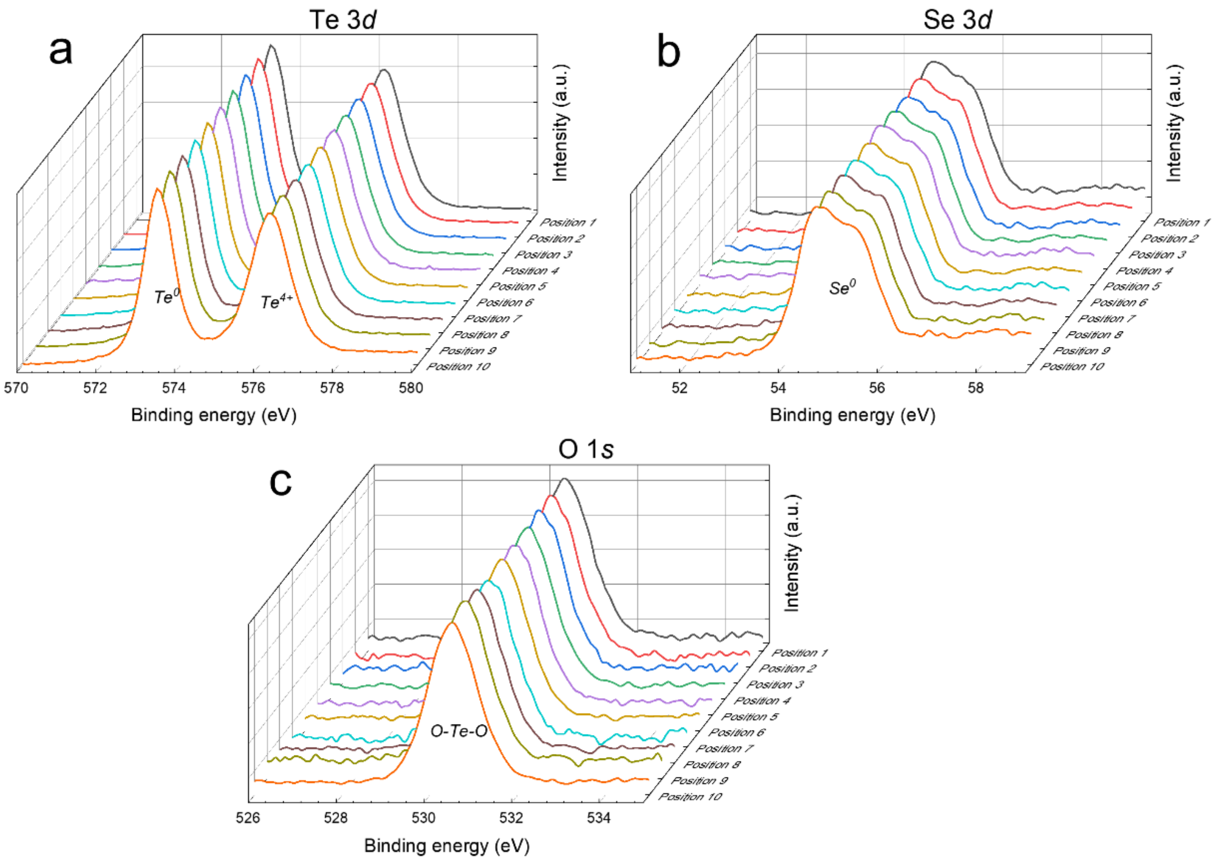

Supplementary Fig. 4. XPS (a) Te 3*d*, (b) O 1*s*, and (c) Se 3*d* analysis of ten different positions of TeSeO thin films grown on a 4-inch SiO_2/Si wafer.

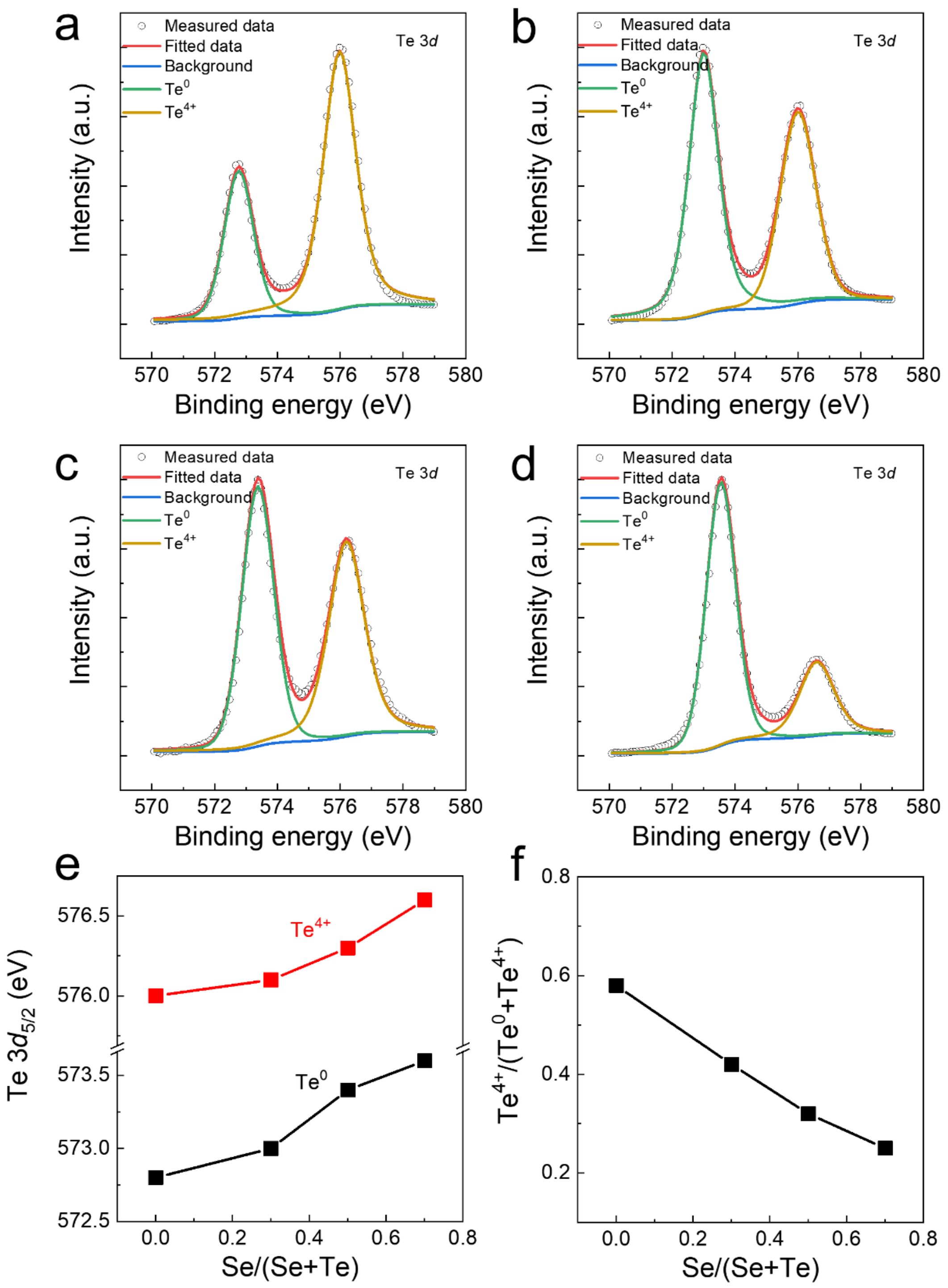

Supplementary Fig. 5. XPS Te 3*d* analysis of (a) $TeO_{1.16}$, (b) $Te_{0.7}Se_{0.3}O_{0.59}$, (c) $Te_{0.5}Se_{0.5}O_{0.32}$, and (d) $Te_{0.3}Se_{0.7}O_{0.15}$ thin films. (e) XPS Te 3$d_{5/2}$ peak analysis of TeSeO thin films with different compositions. (f) Extracted $Te^{4+}/(Te^0+Te^{4+})$ ratios of TeSeO thin films. The coexisted Te^{4+} and Te^0 peaks are clearly distinguished at ~572.8 eV and ~576.0 eV, respectively, which means the partial oxidation of Te. With increasing Se content, the corresponding $Te^{4+}/(Te^0+Te^{4+})$ ratios decrease from ~58% ($TeO_{1.16}$) to ~25% ($Te_{0.3}Se_{0.7}O_{0.59}$), revealing that the Se content can suppress the binding process between Te and O.

Supplementary Table 1. Summary of composition ratios of TeSeO films and mole ratios between Te^0 and Te^{4+}.

**Sample compositions**	**Mole ratios (mol%)**	
	Te^0	Te^{4+}
**$TeO_{1.16}$**	42	58
**$Te_{0.9}Se_{0.1}O_{0.98}$**	45	55
**$Te_{0.8}Se_{0.2}O_{0.80}$**	50	50
**$Te_{0.7}Se_{0.3}O_{0.59}$**	58	42
**$Te_{0.6}Se_{0.3}O_{0.44}$**	63	37
**$Te_{0.5}Se_{0.5}O_{0.32}$**	68	32
**$Te_{0.4}Se_{0.6}O_{0.23}$**	71	29
**$Te_{0.3}Se_{0.7}O_{0.15}$**	75	25
**$Te_{0.2}Se_{0.8}O_{0.09}$**	77	23
**$Te_{0.1}Se_{0.9}O_{0.04}$**	80	20
**Se**	0	0

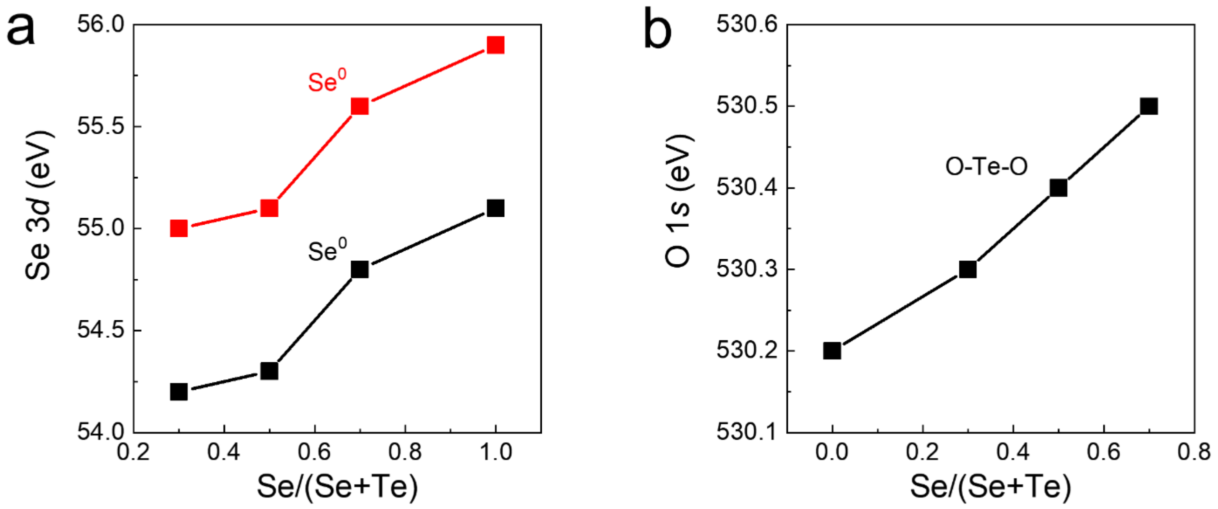

Supplementary Fig. 6. XPS (a) Se 3*d* and (b) O 1*s* peak analysis of TeSeO thin films with different compositions. The distinct peaks observed around 530.2 eV in the O 1*s* spectra imply the O acts as lattice oxygen species O-Te-O. Generally, the adsorbed oxygen or hydroxyl group has higher binding energy, around 532 eV, which is not witnessed in our TeSeO films. At the same time, no Se^{4+} (typically around 60 eV) peak is found from Se 3*d* spectra, mainly owing to its larger electronegativity (2.55) than that of Te (2.1), which makes it difficult to react with oxygen molecules to form SeO_2.

Supplementary Table 2. Summary of the Hall mobilities and hole concentrations of TeSeO thin films.

Material	Hall mobility ($cm^2/(Vs)$)	hole concentration (cm^{-3})
$Te_{0.9}Se_{0.1}O_{0.98}$	60.8	1.1×10^{18}
$Te_{0.8}Se_{0.2}O_{0.8}$	45.2	8.2×10^{17}
$Te_{0.7}Se_{0.3}O_{0.59}$	20.5	4.0×10^{17}

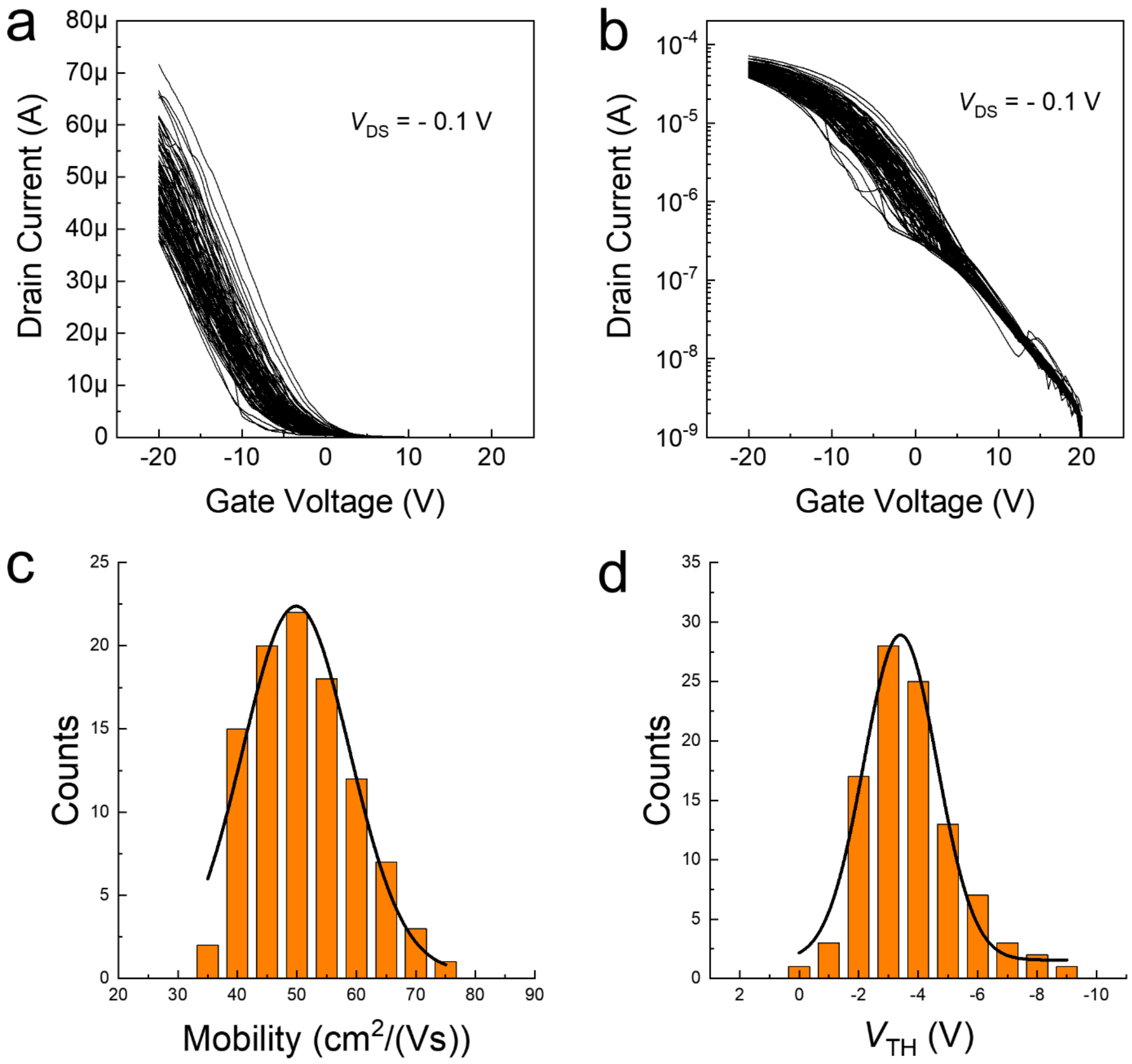

Supplementary Fig. 7. Transfer characteristics of wafer-scale $Te_{0.8}Se_{0.2}O_{0.8}$ TFT array (10×10 array) using (a) linear y-coordinate and (b) logarithm y-coordinate. The corresponding performance distribution of (c) mobility and (d) V_{TH} for wafer-scale $Te_{0.8}Se_{0.2}O_{0.8}$ TFT array. The TFT array shows 100% device yield with hole mobility of 48.2 ± 8.4 cm^2/(Vs), I_{on}/I_{off} of 10^4~10^5, and V_{TH} of -4.2±1.3 V.

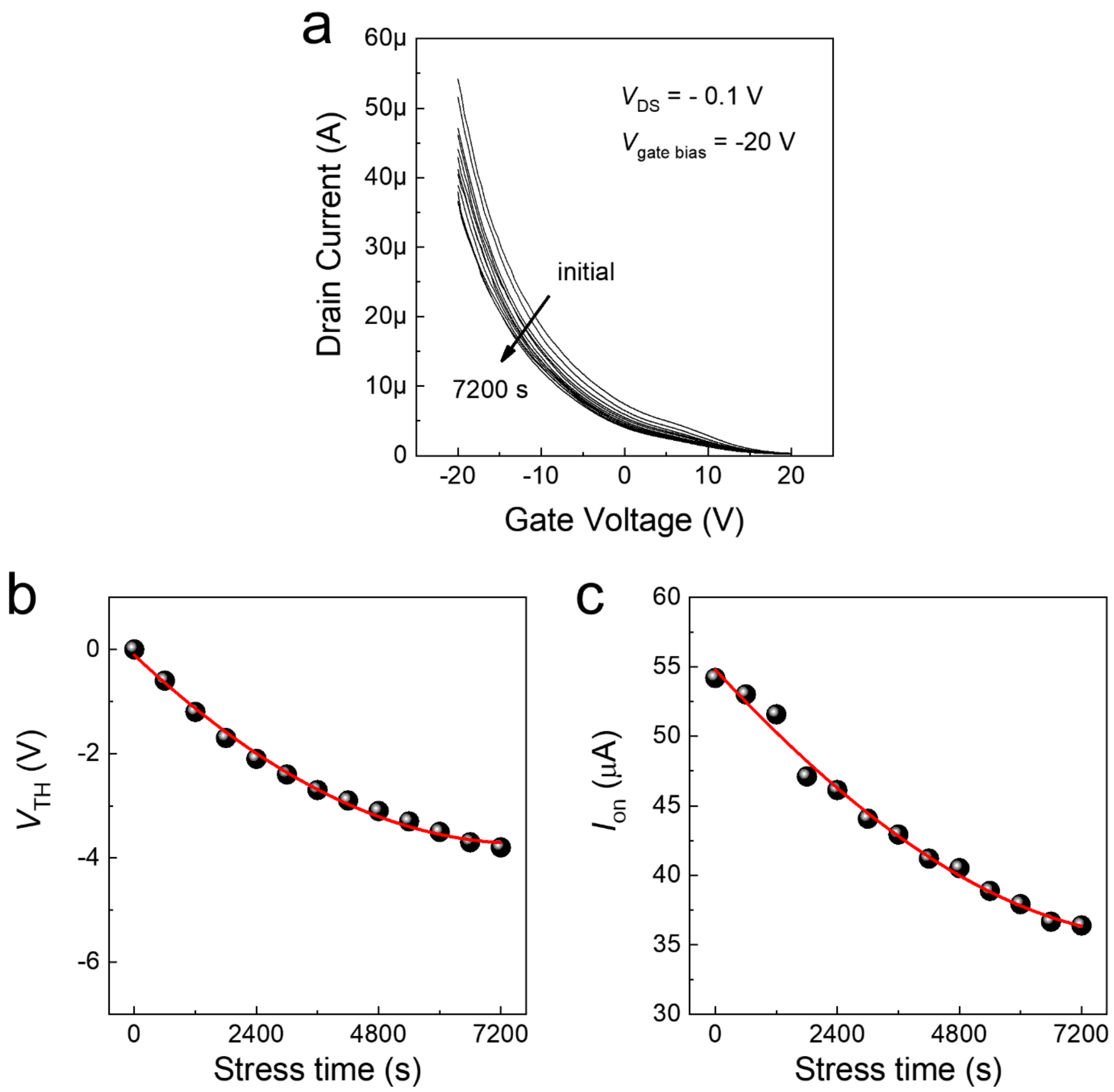

Supplementary Fig. 8. (a) Negative-bias stress (NBS) test of $Te_{0.8}Se_{0.2}O_{0.8}$ TFT. Extracted (b) threshold voltages and (c) on currents of $Te_{0.8}Se_{0.2}O_{0.8}$ TFT in NBS study. After being gated at -20 V for 2 hours, the corresponding V_{TH} shifted negatively from -5 to -8.8 V without noticeable subthreshold swing variation under NBS.

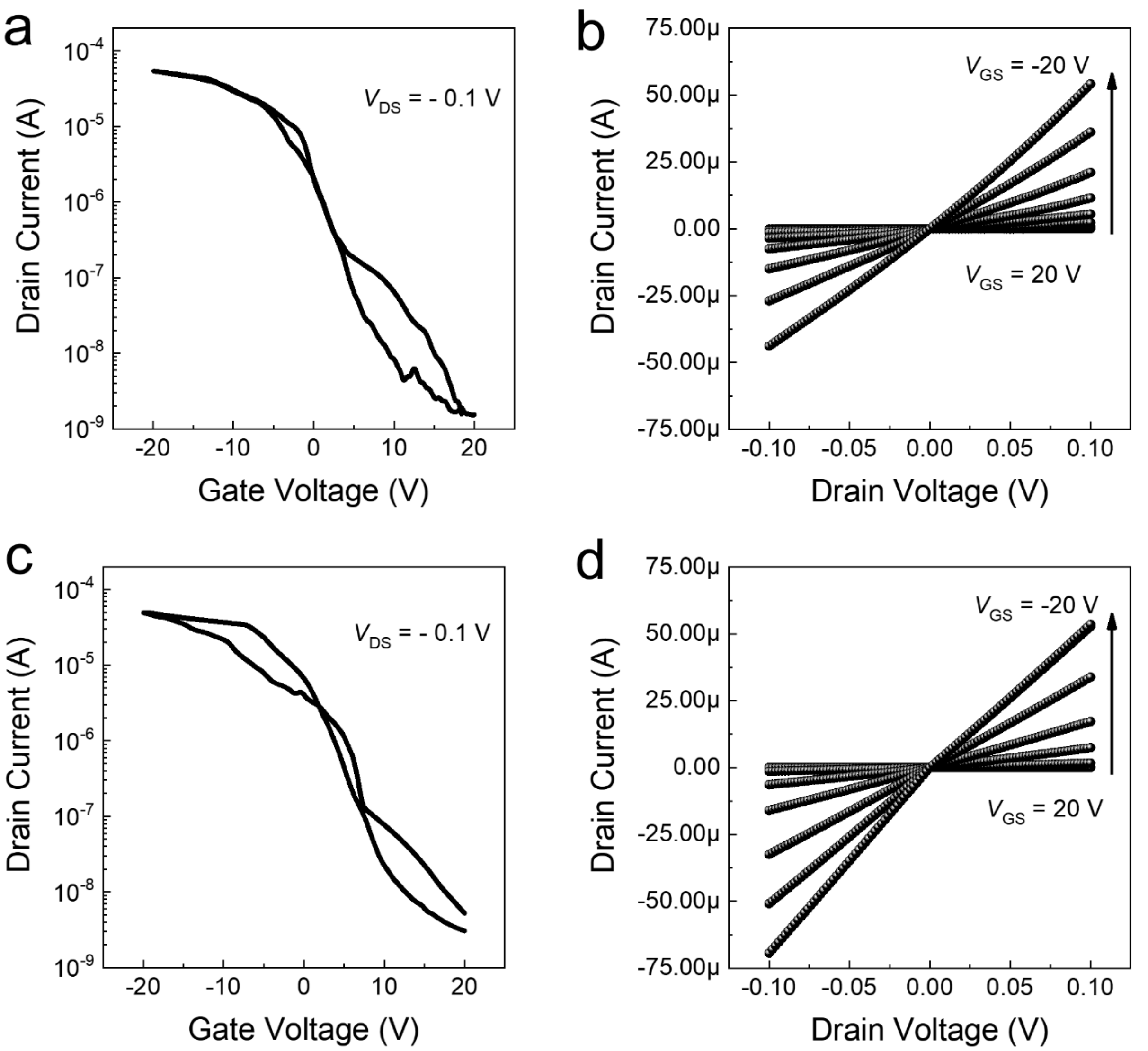

Supplementary Fig. 9. (a) Transfer curve and (b) output curve of $Te_{0.8}Se_{0.2}O_{0.8}$ TFT measured after fabrication. (c) Transfer curve and (d) output curve of $Te_{0.8}Se_{0.2}O_{0.8}$ TFT measured after 300-day storage in ambient. After 300 days of ambient storage, the FET performances are not significantly degraded even without device encapsulation, benefiting from the partial oxidation in TeSeO thin films.

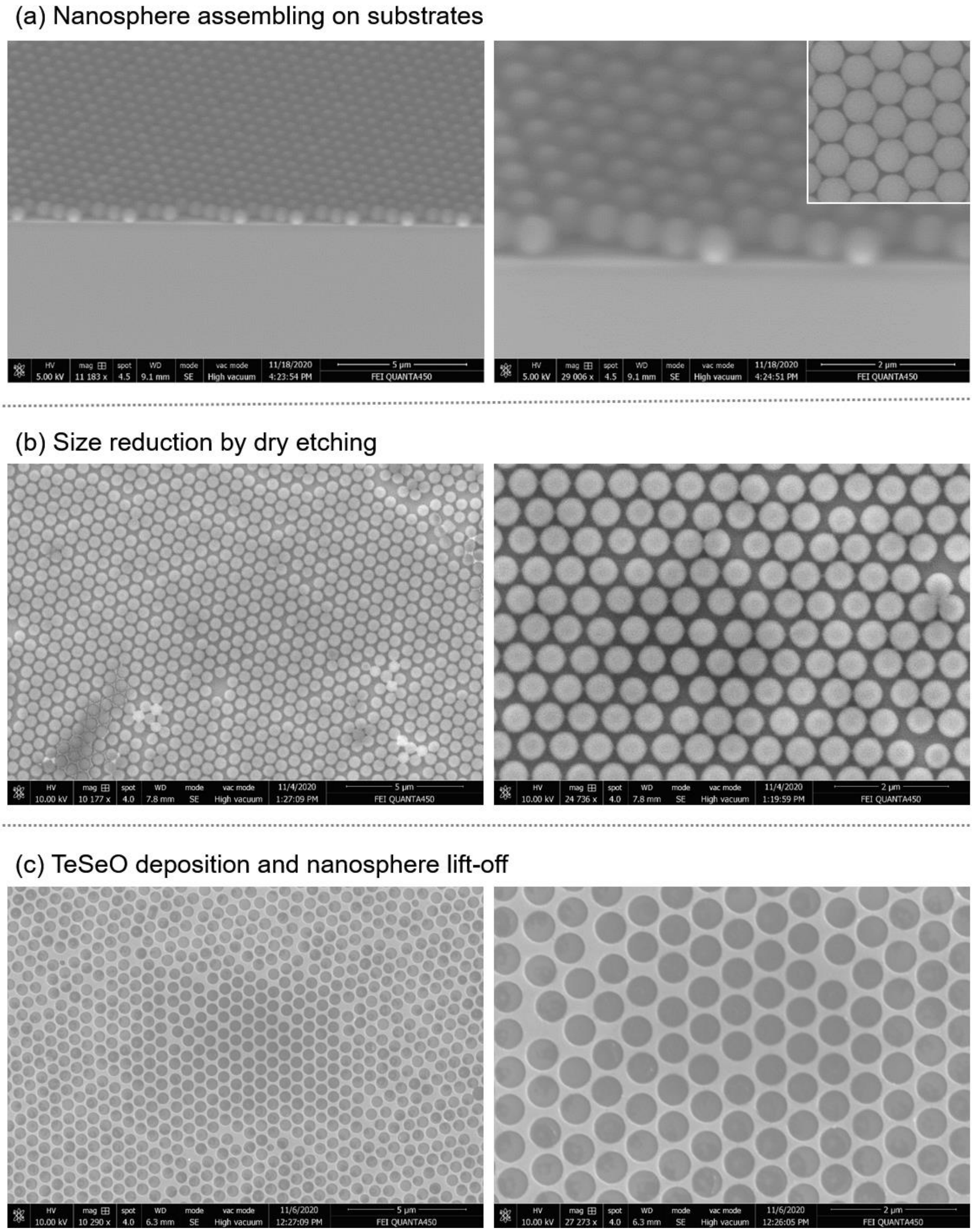

Supplementary Fig. 10. (a) SEM images of the nanosphere lithography process that consists of (a) nanosphere assembling, (b) size reduction, (c) TeSeO deposition, and nanosphere lift-off.

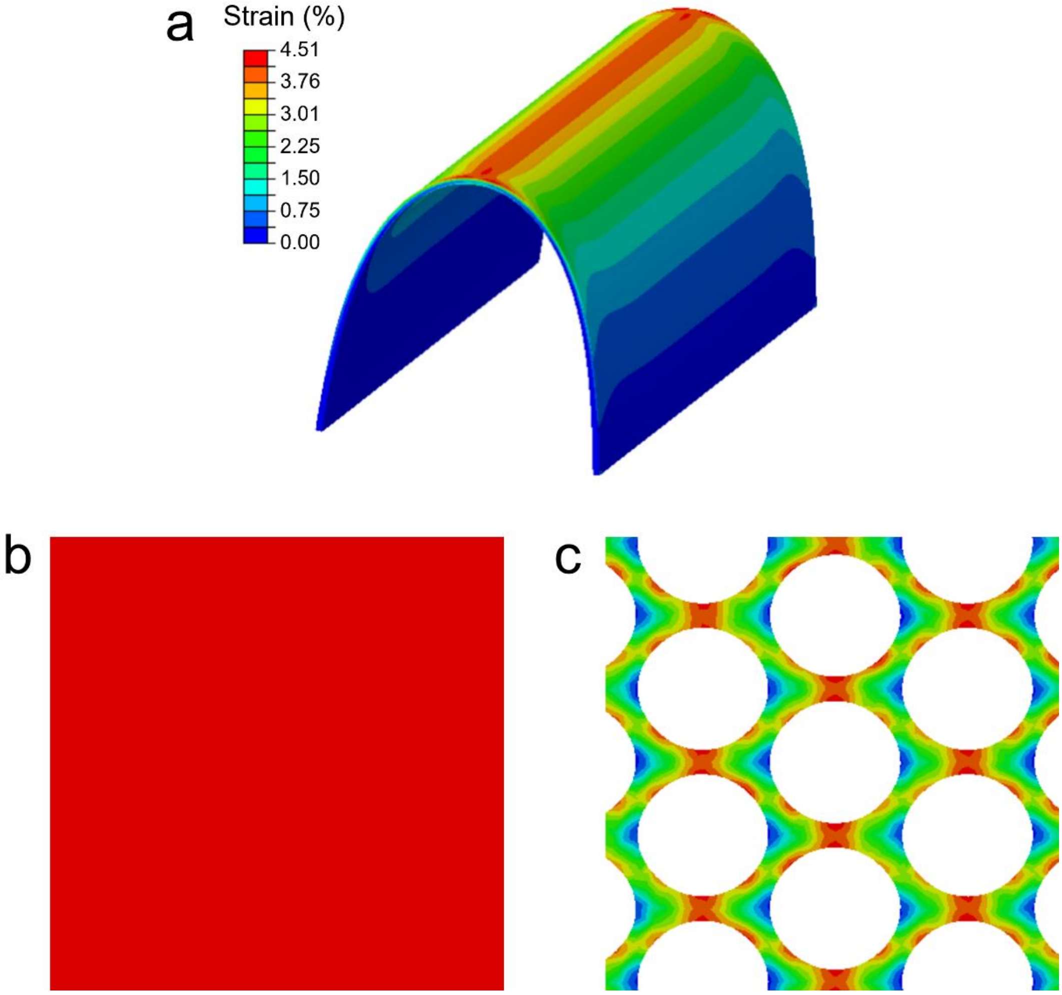

Supplementary Fig. 11. (a) FEA simulation of the TeSeO layer/PI substrate model at a bending radius of 1.5 mm. (b) Top view of strain distribution on the bent TeSeO flat film. (c) Top view of strain distribution on the bent TeSeO honeycomb layer.

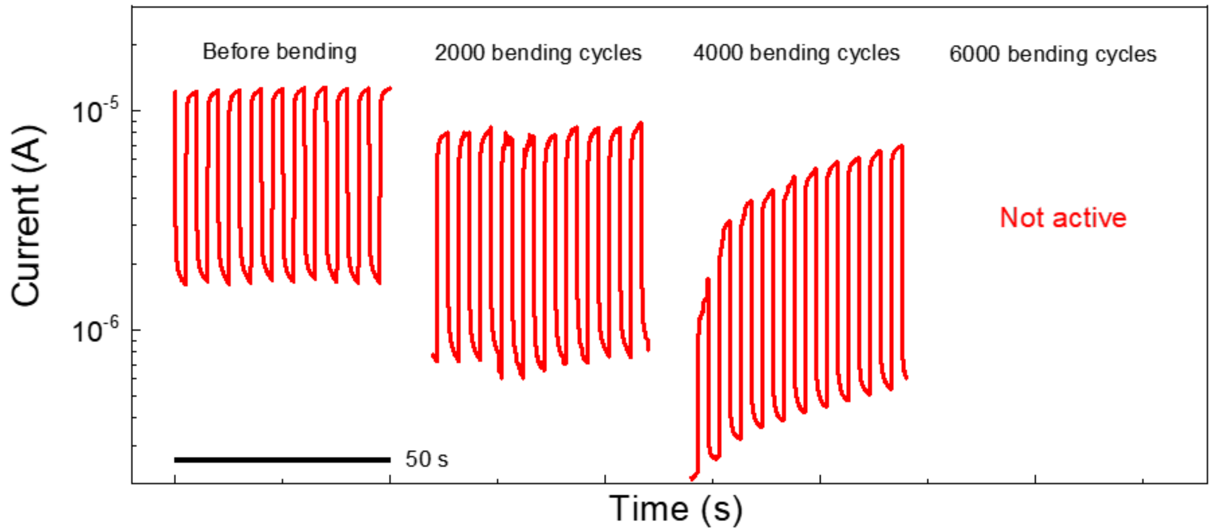

Supplementary Fig. 12. The $Te_{0.7}Se_{0.3}O_{0.59}$ thin film (without nanopatterned) device photocurrent under on/off switching light illumination (0.2 Hz) measured before bending tests, after 2000, 4000, and 6000 bending cycles, respectively.

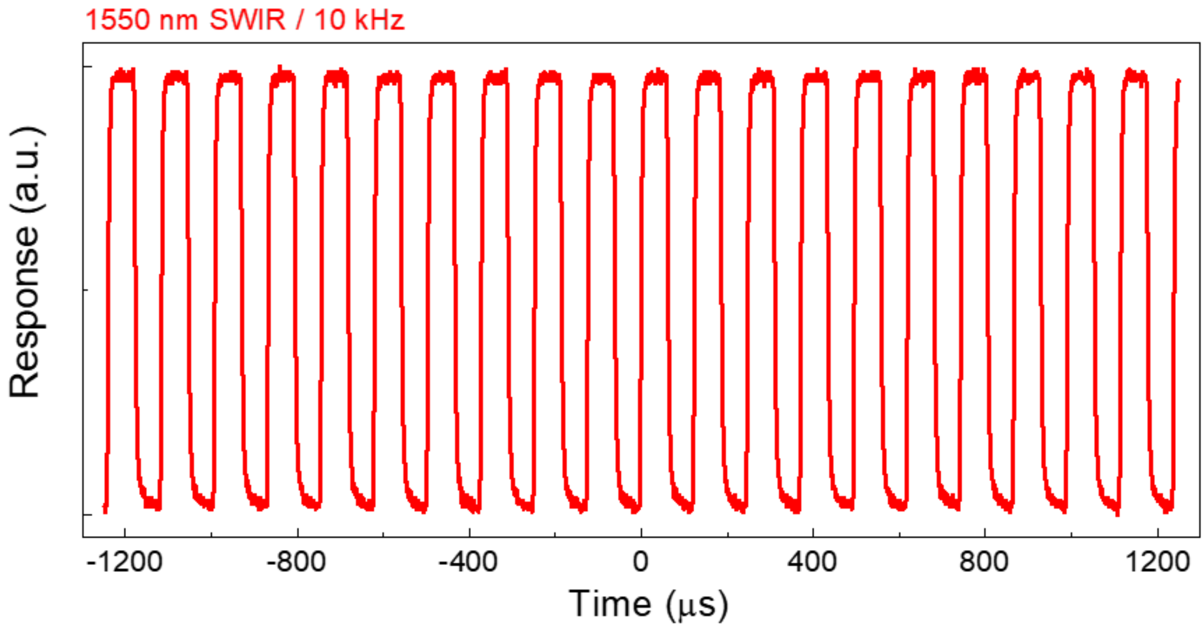

Supplementary Fig. 13. Time-resolved photoresponse of $Te_{0.7}Se_{0.3}O_{0.59}$ honeycomb layer measured at 1550 nm illumination with a chopped frequency of 10 kHz.

Supplementary Table 3. Performance summary of TFTs based on p-type semiconducting thin films, including metal oxides, metal halides, perovskites, organic materials, CNT thin films, and TeSeO.

**Thin-film materials**		**Fabrication temperature**	**Hole mobility [cm²/(Vs)]**	**Reference**
**Metal oxides**	SnO	450 °C	0.13	1
	Cu_2O	700 °C	0.16	2
	Cu_2O	600 °C	0.29	3
	CuO	250 °C	0.30	4
	CuO	220 °C	0.15	5
	NiO	250 °C	0.07	6
	NiO	250 °C	0.48	7
**Metal halides**	CuI	60 °C	1.86	8
	CuI	RT	0.40	9
	CuSnI	140 °C	9	10
	CuBr	RT	0.15	11
	CuI:Zn	80 °C	5.3	12
**Perovskite halides**	$MAPbI_3$	85 °C	2.1	13
	$MAPbI_3$	120 °C	4	14
	$MAPbBr_3$	270 °C	3.6	15
	$MAPbCl_3$	270 °C	3.8	15
	$(PEA)_2SnI_4$	90 °C	15	16
	$(PEA)_2SnI_4$	100 °C	3.5	17
	$CsSnI_3$	120 °C	50	18
**Organic materials**	TIPS-pentacene	90 °C	4.6	19
	C8-BTBT	100 °C	10	20
	C10-DNTT	80 °C	5.8	21
	C10-DNBDT	135 °C	7.2	22
	DPh-DNTT	90 °C	4.3	23
**CNT**	CNT thin films	200 °C	50	24
	CNT thin films	150 °C	9	25
	CNT thin films	125 °C	3.92	26
	CNT thin films	80 °C	12	27
	CNT thin films	140 °C	14	28
**TeSeO**	$Te_{0.7}Se_{0.3}O_{0.59}$	RT	23.1	This work
	$Te_{0.8}Se_{0.2}O_{0.8}$	RT	48.5	This work
	$Te_{0.9}Se_{0.1}O_{0.98}$	RT	65.5	This work

RT = room temperature

Supplementary Table 4. Performance summary of PDs based on Te, perovskite halides, metal oxides, Group III-Vs, layered materials, and TeSeO

Materials		*R* [A/W]	Rise time [µs]	Decay time [µs]	Reference
**Te**	Te nanoplate	162.4	4.4×10^6	2.8×10^6	29
	Te nanosheet	-	1.09×10^6	2.51×10^6	30
	Te@Se NW	1×10^{-4}	9×10^4	1×10^5	31
	Te nanoflake	15	5×10^5	1×10^6	32
	Te nanomesh	~2.5	3×10^5	6×10^5	33
	Te nanobelt	254.2	510	300	34
	Te nanoflake	1360	48.7	62.7	35
**Perovskite halides**	$CsPbBr_3$	0.64	19	24	36
	$CsPbBr_3$	0.18	1800	1000	37
	$MAPbI_3$	13.5	80	240	38
	$MAPbI_3$	4.95	100	100	39
	$CsPbBr_3$	34	600	900	40
	$CsPbBr_3$	31.1	16	380	41
	$MAPbI_3$	410	220	790	42
	$CsPbCl_3$	1183	5×10^4	5×10^4	43
**Metal oxides**	InGaZnO	1.95×10^6	9.3×10^5	2×10^5	44
	Ga_2O_3	547	1×10^6	6×10^5	45
	Ga_2O_3	3000	1×10^5	3×10^4	46
	CuO	15.3	6.8×10^5	1.77×10^6	47
	$ZnGa_2O_4$	474	4×10^5	7×10^5	48
	ZnO	6830	3×10^5	3.1×10^5	49
	ZnSnO	11.3	1.78×10^6	3.02×10^6	50
**Group III-Vs**	InGaSb	1520	13	16	51
	InGaSb	6000	38	53	52
	InAs	-	1.2×10^4	6×10^3	53
	InGaAs	7300	4.8×10^5	8.1×10^5	54
	GaSb	61	1.95×10^5	3.8×10^5	55
	InSb	311.5	4.2×10^6	5.5×10^6	56
	InAs	4400	1400	600	57
**Layered materials**	WS_2	0.0032	1.6×10^5	1.7×10^5	58
	$PdSe_2$	42.1	7.45×10^4	9.31×10^4	59
	Bi_2O_2Se	6.5	2800	4600	60
	$PtTe_2$	0.005	2.4	32	61
	b-PAs	16	12.4	8.6	62
	InSe	274	1.51×10^4	6.37×10^4	63
	MoS_2	12	10	20	64
**TeSeO**		603	5	7	This work

Supplementary References

1 Okamura, K., Nasr, B., Brand, R. A. & Hahn, H. Solution-processed oxide semiconductor SnO in p-channel thin-film transistors. *J. Mater. Chem.* **22**, 4607-4610 (2012).

2 Kim, S. Y. *et al.* p-Channel oxide thin film transistors using solution-processed copper oxide. *ACS applied materials interfaces* **5**, 2417-2421 (2013).

3 Yu, J. *et al.* Solution-processed p-type copper oxide thin-film transistors fabricated by using a one-step vacuum annealing technique. *Journal of Materials Chemistry C* **3**, 9509-9513 (2015).

4 Liu, A. *et al.* In situ one-step synthesis of p-type copper oxide for low-temperature, solution-processed thin-film transistors. *Journal of Materials Chemistry C* **5**, 2524-2530 (2017).

5 Liu, A., Zhu, H. & Noh, Y.-Y. Polyol Reduction: A Low-Temperature Eco-Friendly Solution Process for p-Channel Copper Oxide-Based Transistors and Inverter Circuits. *ACS applied materials interfaces* **11**, 33157-33164 (2019).

6 Liu, A. *et al.* Hole mobility modulation of solution-processed nickel oxide thin-film transistor based on high-k dielectric. *Appl. Phys. Lett.* **108**, 233506 (2016).

7 Xu, W. *et al.* p-Type transparent amorphous oxide thin-film transistors using low-temperature solution-processed nickel oxide. *Journal of Alloys Compounds* **806**, 40-51 (2019).

8 Choi, C.-H. *et al.* Low-temperature, inkjet printed p-type copper (I) iodide thin film transistors. *Journal of Materials Chemistry C* **4**, 10309-10314 (2016).

9 Liu, A. *et al.* Room-Temperature Solution-Synthesized p-Type Copper (I) Iodide Semiconductors for Transparent Thin-Film Transistors and Complementary Electronics. *Adv. Mater.* **30**, 1802379 (2018).

10 Jun, T., Kim, J., Sasase, M. & Hosono, H. Material Design of p-Type Transparent Amorphous Semiconductor, Cu–Sn–I. *Adv. Mater.* **30**, 1706573 (2018).

11 Zhu, H., Liu, A. & Noh, Y.-Y. Transparent Inorganic Copper Bromide (CuBr) p-Channel Transistors Synthesized From Solution at Room Temperature. *IEEE Electron Device Letters* **40**, 769-772 (2019).

12 Liu, A. *et al.* High-performance p-channel transistors with transparent Zn doped-CuI. *Nat. Commun.* **11**, 4309 (2020).

13 Cho, N. *et al.* Pure crystal orientation and anisotropic charge transport in large-area hybrid perovskite films. *Nat. Commun.* **7**, 13407, doi:10.1038/ncomms13407 (2016).

14 Li, D. *et al.* The Effect of Thermal Annealing on Charge Transport in Organolead Halide Perovskite Microplate Field-Effect Transistors. *Adv. Mater.* **29**, 1601959, doi:10.1002/adma.201601959 (2017).

15 Yu, W. *et al.* Single crystal hybrid perovskite field-effect transistors. *Nat. Commun.* **9**, 5354, doi:10.1038/s41467-018-07706-9 (2018).

16 Matsushima, T. *et al.* Solution-Processed Organic-Inorganic Perovskite Field-Effect Transistors with High Hole Mobilities. *Adv. Mater.* **28**, 10275-10281, doi:10.1002/adma.201603126 (2016).

17 Zhu, H. *et al.* High-Performance and Reliable Lead-Free Layered-Perovskite Transistors. *Adv. Mater.* **32**, 2002717 (2020).

18 Liu, A. *et al.* High-performance inorganic metal halide perovskite transistors. *Nat. Electron.* **5**, 78-83, doi:10.1038/s41928-022-00712-2 (2022).

19 Giri, G. *et al.* Tuning charge transport in solution-sheared organic semiconductors using lattice strain. *Nature* **480**, 504-508 (2011).

20 He, D. *et al.* Two-dimensional quasi-freestanding molecular crystals for high-performance organic field-effect transistors. *Nat. Commun.* **5**, 1-7 (2014).

21 Uemura, T. *et al.* On the Extraction of Charge Carrier Mobility in High-Mobility Organic Transistors. *Adv. Mater.* **28**, 151-155 (2016).

22 Rolin, C. *et al.* Charge carrier mobility in thin films of organic semiconductors by the gated van der Pauw method. *Nat. Commun.* **8**, 1-9 (2017).

23 Borchert, J. W. *et al.* Flexible low-voltage high-frequency organic thin-film transistors. *Sci. Adv.* **6**, eaaz5156 (2020).

24 Wang, C. *et al.* Extremely bendable, high-performance integrated circuits using semiconducting carbon nanotube networks for digital, analog, and radio-frequency applications. *Nano Lett.* **12**, 1527-1533 (2012).

25 Lau, P. H. *et al.* Fully printed, high performance carbon nanotube thin-film transistors on flexible substrates. *Nano Lett.* **13**, 3864-3869 (2013).

26 Cao, X. *et al.* Fully screen-printed, large-area, and flexible active-matrix electrochromic displays using carbon nanotube thin-film transistors. *ACS Nano* **10**, 9816-9822 (2016).

27 Cardenas, J. A. *et al.* In-place printing of carbon nanotube transistors at low temperature. *ACS Applied Nano Materials* **1**, 1863-1869 (2018).

28 Zhang, Z., Du, C., Jiao, H. & Zhang, M. Polyvinyl Alcohol/SiO_2 Hybrid Dielectric for Transparent Flexible/Stretchable All-Carbon-Nanotube Thin-Film-Transistor Integration. *Advanced Electronic Materials* **6**, 1901133 (2020).

29 Wang, Q. *et al.* Van der Waals epitaxy and photoresponse of hexagonal tellurium nanoplates on flexible mica sheets. *ACS Nano* **8**, 7497-7505 (2014).

30 Peng, J. *et al.* Two-Dimensional Tellurium Nanosheets Exhibiting an Anomalous Switchable Photoresponse with Thickness Dependence. *Angew. Chem. Int. Ed.* **57**, 13533-13537 (2018).

31 Huang, W. *et al.* Enhanced Photodetection Properties of Tellurium@ Selenium Roll-to-Roll Nanotube Heterojunctions. *Small* **15**, 1900902 (2019).

32 Zhang, X. *et al.* Hydrogen-Assisted Growth of Ultrathin Te Flakes with Giant Gate-Dependent Photoresponse. *Adv. Funct. Mater.* **29**, 1906585 (2019).

33 Dang, S. *et al.* Piezoelectric modulation of broadband photoresponse of flexible tellurium nanomesh photodetectors. *Nanotechnology* **31**, 095502 (2019).

34 Kang, S. *et al.* Broad spectral response of an individual tellurium nanobelt grown by molecular beam epitaxy. *Nanoscale* **11**, 1879-1886 (2019).

35 Tong, L. *et al.* Stable mid-infrared polarization imaging based on quasi-2D tellurium at room temperature. *Nat. Commun.* **11**, 2308 (2020).

36 Song, J. *et al.* Monolayer and Few-Layer All-Inorganic Perovskites as a New Family of Two-Dimensional Semiconductors for Printable Optoelectronic Devices. *Adv. Mater.* **28**, 4861-4869, (2016).

37 Li, X. *et al.* Healing All-Inorganic Perovskite Films via Recyclable Dissolution-Recyrstallization for Compact and Smooth Carrier Channels of Optoelectronic Devices with High Stability. *Adv. Funct. Mater.* **26**, 5903-5912, (2016).

38 Deng, W. *et al.* Aligned Single-Crystalline Perovskite Microwire Arrays for High-Performance Flexible Image Sensors with Long-Term Stability. *Adv. Mater.* **28**, 2201-2208, (2016).

39 Gao, L. *et al.* Passivated Single-Crystalline $CH_3NH_3PbI_3$ Nanowire Photodetector with High Detectivity and Polarization Sensitivity. *Nano Lett.* **16**, 7446-7454, (2016).

40 Liu, X. *et al.* Low-Voltage Photodetectors with High Responsivity Based on Solution-Processed Micrometer-Scale All-Inorganic Perovskite Nanoplatelets. *Small* **13**, 1700364, (2017).
41 Li, X. *et al.* Constructing Fast Carrier Tracks into Flexible Perovskite Photodetectors To Greatly Improve Responsivity. *ACS Nano* **11**, 2015-2023, (2017).
42 Zhou, Q. *et al.* Nanochannel-Assisted Perovskite Nanowires: From Growth Mechanisms to Photodetector Applications. *ACS Nano* **12**, 8406-8414, (2018).
43 Meng, Y. *et al.* Direct Vapor–Liquid–Solid Synthesis of All-Inorganic Perovskite Nanowires for High-Performance Electronics and Optoelectronics. *ACS Nano* **13**, 6060-6070, (2019).
44 Li, F. *et al.* High-Performance Transparent Ultraviolet Photodetectors Based on InGaZnO Superlattice Nanowire Arrays. *ACS Nano* **13**, 12042-12051, (2019).
45 Tian, W. *et al.* In-doped Ga_2O_3 nanobelt based photodetector with high sensitivity and wide-range photoresponse. *J. Mater. Chem.* **22**, 17984-17991 (2012).
46 Qin, Y. *et al.* Enhancement-Mode β-Ga_2O_3 Metal–Oxide–Semiconductor Field-Effect Solar-Blind Phototransistor With Ultrahigh Detectivity and Photo-to-Dark Current Ratio. *IEEE Electron Device Letters* **40**, 742-745 (2019).
47 Song, H.-J. *et al.* High-performance copper oxide visible-light photodetector via grain-structure model. *Scientific Reports* **9**, 7334 (2019).
48 Huang, P.-H. *et al.* Energy-Saving $ZnGa_2O_4$ Phototransistor Improved by Thermal Annealing. *ACS Applied Electronic Materials* **2**, 3515-3521 (2020).
49 Wang, Y. *et al.* High performance charge-transfer induced homojunction photodetector based on ultrathin ZnO nanosheet. *Appl. Phys. Lett.* **114**, 011103 (2019).
50 Huang, C.-Y. *et al.* High-performance solution-processed ZnSnO metal–semiconductor–metal ultraviolet photodetectors via ultraviolet/ozone photo-annealing. *Semiconductor Science Technology* **36**, 095013 (2021).
51 Li, D. *et al.* Flexible Near-Infrared InGaSb Nanowire Array Detectors with Ultrafast Photoconductive Response Below 20 μs. *Adv. Opt. Mater.* **8**, 2001201 (2020).
52 Li, D. *et al.* Ultra-fast photodetectors based on high-mobility indium gallium antimonide nanowires. *Nat. Commun.* **10**, 1664 (2019).
53 Guo, N. *et al.* Anomalous and highly efficient InAs nanowire phototransistors based on majority carrier transport at room temperature. *Adv. Mater.* **26**, 8203-8209 (2014).
54 Zhang, H. *et al.* Enhanced performance of near-infrared photodetectors based on InGaAs nanowires enabled by a two-step growth method. *Journal of Materials Chemistry C* **8**, 17025-17033 (2020).
55 Sun, J. *et al.* Ultrahigh hole mobility of Sn-catalyzed GaSb nanowires for high speed infrared photodetectors. *Nano Lett.* **19**, 5920-5929 (2019).
56 Zhang, S. *et al.* Highly sensitive InSb nanosheets infrared photodetector passivated by ferroelectric polymer. *Adv. Funct. Mater.* **30**, 2006156 (2020).
57 Zhang, X. *et al.* Surface-states-modulated high-performance InAs nanowire phototransistor. *The Journal of Physical Chemistry Letters* **11**, 6413-6419 (2020).
58 Lan, C. *et al.* Utilizing a NaOH promoter to achieve large single-domain monolayer WS_2 films via modified chemical vapor deposition. *ACS applied materials interfaces* **11**, 35238-35246 (2019).
59 Long, M. *et al.* Palladium diselenide long-wavelength infrared photodetector with high sensitivity and stability. *ACS Nano* **13**, 2511-2519 (2019).

60 Li, J. *et al.* High-performance near-infrared photodetector based on ultrathin Bi_2O_2Se nanosheets. *Adv. Funct. Mater.* **28**, 1706437 (2018).
61 Zeng, L. *et al.* Van der Waals epitaxial growth of mosaic-like 2D platinum ditelluride layers for room-temperature mid-infrared photodetection up to 10.6 μm. *Adv. Mater.* **32**, 2004412 (2020).
62 Amani, M., Regan, E., Bullock, J., Ahn, G. H. & Javey, A. Mid-wave infrared photoconductors based on black phosphorus-arsenic alloys. *ACS Nano* **11**, 11724-11731 (2017).
63 Curreli, N. *et al.* Liquid phase exfoliated indium selenide based highly sensitive photodetectors. *Adv. Funct. Mater.* **30**, 1908427 (2020).
64 Lv, L. *et al.* Reconfigurable two-dimensional optoelectronic devices enabled by local ferroelectric polarization. *Nat. Commun.* **10**, 1-10 (2019).
